# IL-35 expression in hepatocellular carcinoma cells is associated with tumor progression

**DOI:** 10.18632/oncotarget.10141

**Published:** 2016-06-17

**Authors:** Jun Long, Hongyan Guo, Shichang Cui, Haiyan Zhang, Xinmin Liu, Danning Li, Zimeng Han, Linfeng Xi, Wenyi Kou, Jiangnan Xu, Tao-Sheng Li, Yaozhong Ding

**Affiliations:** ^1^ Department of Immunology, School of Basic Medical Sciences, Capital Medical University, Beijing, 100069, P.R. China; ^2^ Clinical Laboratory of Beijing Youan Hospital, Capital Medical University, Beijing, 100069, P.R. China; ^3^ Oncology and Hepatobiliary Minimally Invasive Interventional Center, Beijing Youan Hospital, Capital Medical University, Beijing, 100069, P.R. China; ^4^ Department of Cell Biology, Municipal Laboratory for Liver Protection and Regulation of Regeneration, Capital Medical University, Beijing, 100069, P.R. China; ^5^ Department of Stem Cell Biology, Atomic Bomb Disease Institute, Nagasaki University, Nagasaki, 852-8523, Japan

**Keywords:** IL-35, hepatocellular carcinoma, tumor progression, migration, invasion

## Abstract

IL-35 has recently been demonstrated to play significant roles in the progression of various malignant tumors. We investigated the expression of IL-35 in hepatocellular carcinoma (HCC) and the regulatory mechanisms in HCC progression. Tissue microarray from 75 HCC patients revealed that IL-35 was primarily localized in the cytoplasm of cancer cells and peri-tumoral hepatocytes. Quantitative analysis showed that IL-35 expression was significantly lower in patients in the advanced stages than in the early stages. Significantly lower expression of IL-35 was also observed in HCC patients with higher histological grades, larger tumor size, positive microvascular invasion and lymph node/distant metastasis. IL-35 over-expression in HepG2 cells significantly upregulated HLA-ABC and CD95, reduced activities of MMP-2 and MMP-9, and decreased cell migration, invasion and colony formation capacities. Our data indicated that decreased expression of IL-35 in tumor tissues might contribute to the progression of HCC, and IL-35 may serve as a new therapeutic target for HCC.

## INTRODUCTION

Interleukin 35 (IL-35) is a heterodimeric cytokine of the IL-12 family [1], mainly expressed in immune cells including the regulatory T cells (T_regs_) [2–4], regulatory B cells (B_regs_) [5–6], and plasma cells [7]. As an anti-inflammatory and immune inhibitory cytokine, IL-35 has been demonstrated to suppress T cell proliferation [1], convert naive T cells into IL-35-producing iTr35 [8], and play important roles in inflammatory diseases [9–10], infectious diseases [5, 11–12], autoimmune diseases [13–16], and allograft rejection [17–18].

Interestingly, the positive expression of IL-35 has recently been observed in various malignant tumors [19–24], such as lung cancer [19], pancreatic cancer [20], colorectal cancer [21], nasopharyngeal carcinoma [22] and gastric cancer [24]. It has also been demonstrated that the expression of IL-35 in cancer cells is involved in tumor development and prognosis, presumably due to its immune inhibitory activities. However, the precise role of IL-35 expression in cancer cells remains to be revealed, and we have limited information about the IL-35 expression in hepatocellular carcinoma (HCC), one of the most common types of cancer worldwide [25–26].

In this study, we examined the expression of IL-35 in different AJCC TNM stages of HCC using a tissue microarray and further analyzed whether IL-35 expression levels were associated with the clinicopathological features of HCC patients. To further confirm the role of IL-35 expression in the progression of HCC, we used HepG2 cells (a cell line of HCC) with IL-35 over-expression to investigate how IL-35 over-expression could change the biological characteristics of cells.

## RESULTS

### Patients and clinicopathological features

All tissue samples were collected from patients (63 men and 12 women) who underwent precise hepatectomy with a pathologically confirmed diagnosis of primary HCC, and none of the patients had received radiotherapy or chemotherapy before surgery. The clinical and pathological features of these patients are summarized in Table 1. The median age at diagnosis was 53 years old (range 34 to 73 years old). According to the American Joint Committee on Cancer (AJCC, Seventh Edition) guidelines, patients were determined to be stage I in 17 (23%) cases, stage II in 26 (35%) cases, stage III in 22 cases (29%), and stage IV in 10 (13%) cases. Based on the WHO criteria, the histological grade of tumors was defined as grade I in 3 (4%) cases, grade II in 46 (61%) cases, grade III in 25 (33%) cases, and grade IV in 1 (2%) cases.

**Table 1:** clinicopathological features of patients with hepatocellular carcinoma

Clinicopathological variables	No. of patients (%)
Age at diagnosis (34–73 yrs, media: 53 yrs)	
≤ 53	43 (57)
> 53	32 (43)
Gender	
Male	63 (84)
Female	12 (16)
Tumor size	
≤ 5 cm	28 (42)
> 5 cm	38 (58)
Microvascular invasion	
Yes	24 (32)
No	51 (68)
Lymph node metastasis	
positive	5 (7)
negative	70 (93)
Distant metastasis	
positive	5 (7)
negative	70 (93)
Histological grade of tumor	
grade I–II	49 (65)
grade III–IV	26 (35)
AJCC TNM stage	
stage I–II	43 (57)
stage III–IV	32 (43)

### Low IL-35 expression levels in HCC tissues associated with poor prognosis of patients

**Figure 1:**
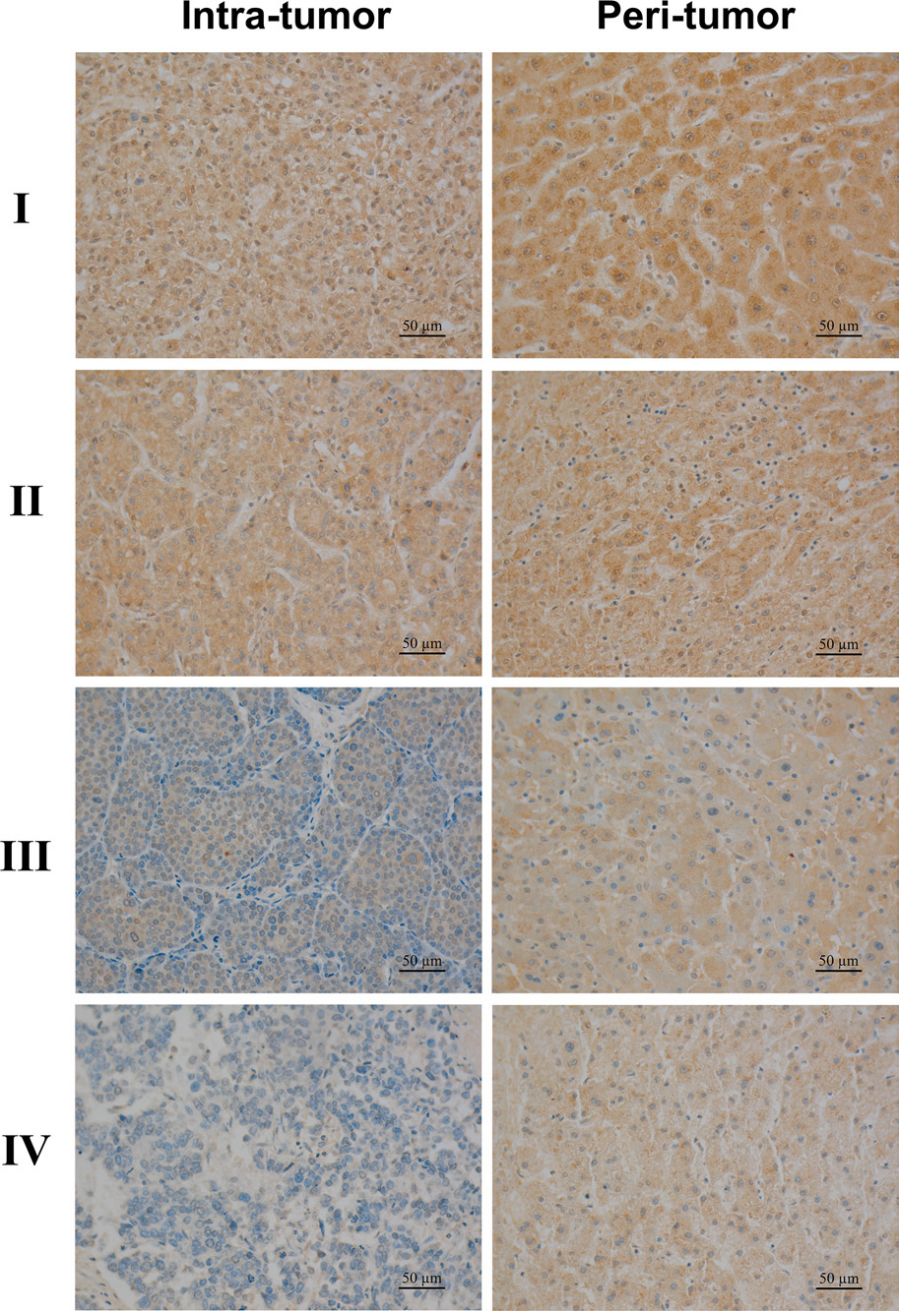
Tissue microarray analysis of IL-35 expression in hepatocellular carcinoma tissues A anti-human IL-35 mAb was used to stain human hepatocellular carcinoma (HCC) tissues and peri-tumoral liver tissues. Representative images of IL-35 expression in tumor and peri-tumoral liver tissues from HCC patients of AJCC TNM stage I (A, B), II (C, D), III (E, F), IV (G, H). Positive staining was observed mainly in the cytoplasm and partially in the nucleus of cancer cells and peri-tumoral hepatocytes, but stroma cells were negatively stained (bar, 50 μm, 400 × magnification).

A tissue microarray containing matched pairs of different stages of primary HCC tissue and adjacent liver tissues from 75 patients was used for immunohistochemical analysis. The expression of IL-35 was observed in tumoral cells and some hepatocytes around the peri-tumoral zone, and positive staining was mainly localized in the cytoplasm and also weakly observed in the nucleus. However, stroma cells were negative for IL-35 staining (Figure 1A–1H).

**Figure 2:**
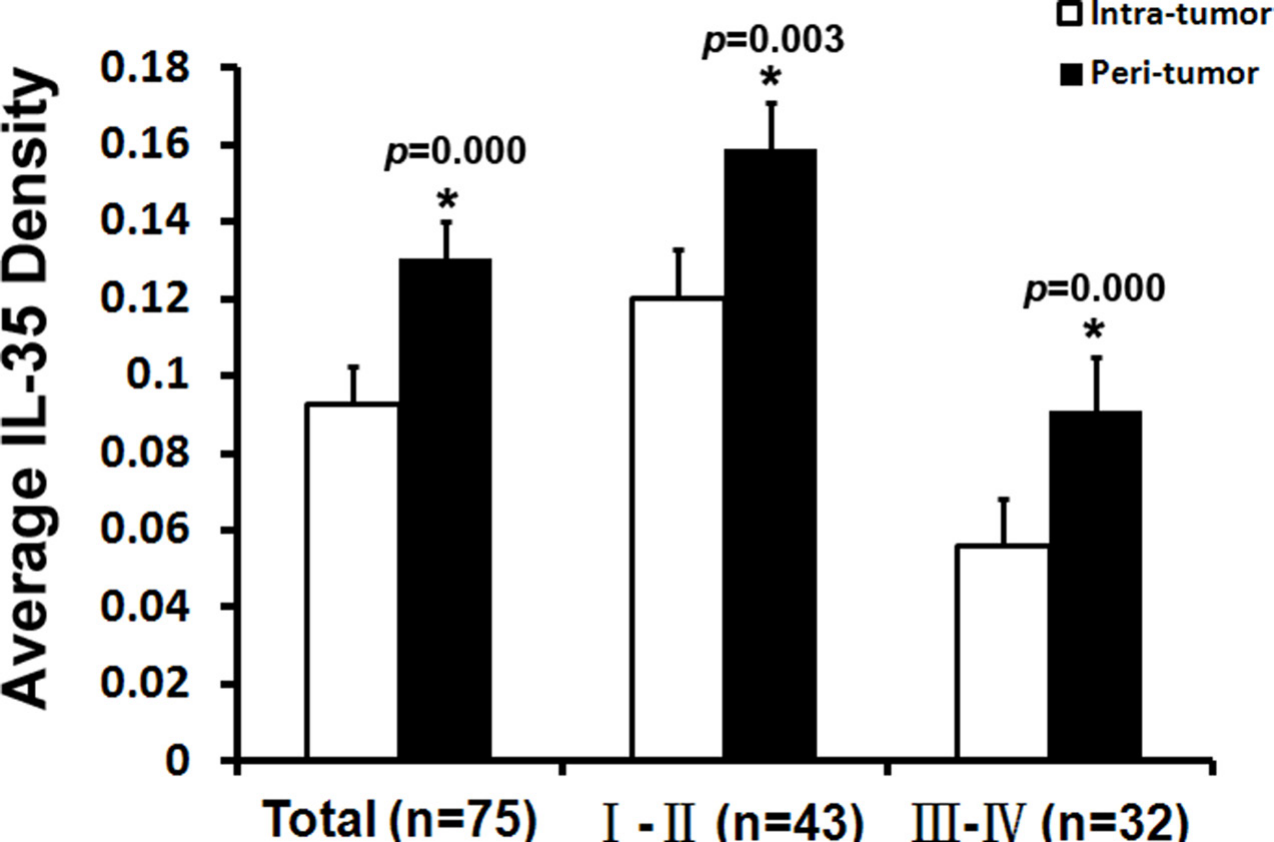
Average density of IL-35 staining in tumor tissue and peri-tumoral liver tissues Semi-quantification of the IL-35 expression was performed by measuring the density of positive staining. IL-35 densities were significantly higher in the peri-tumoral liver tissue than the intra-tumoral zone, and the same situation occurred for the early stage (AJCC TNM stage I-II) and advanced stage (AJCC TNM stage III-IV). Data are expressed as the mean ± SEM. (*) The paired samples *t*-test showed a significant difference between the two groups.

We performed semi-quantification of the IL-35 expression by measuring the density of positive staining. Interestingly, the expression levels of IL-35 were significantly higher in the peri-tumoral zone than the intra-tumoral zone (0.130 ± 0.010 vs. 0.093 ± 0.010, *p* = 0.000), and the same situation occurred in the early stages (including AJCC TNM stage I-II, 0.159 ± 0.012 vs. 0.120 ± 0.013, *p* = 0.003) and advanced stages (including AJCC TNM stage III-IV (0.091 ± 0.014 vs. 0.056 ± 0.012, *p* = 0.000) (Figure 2).

**Figure 3:**
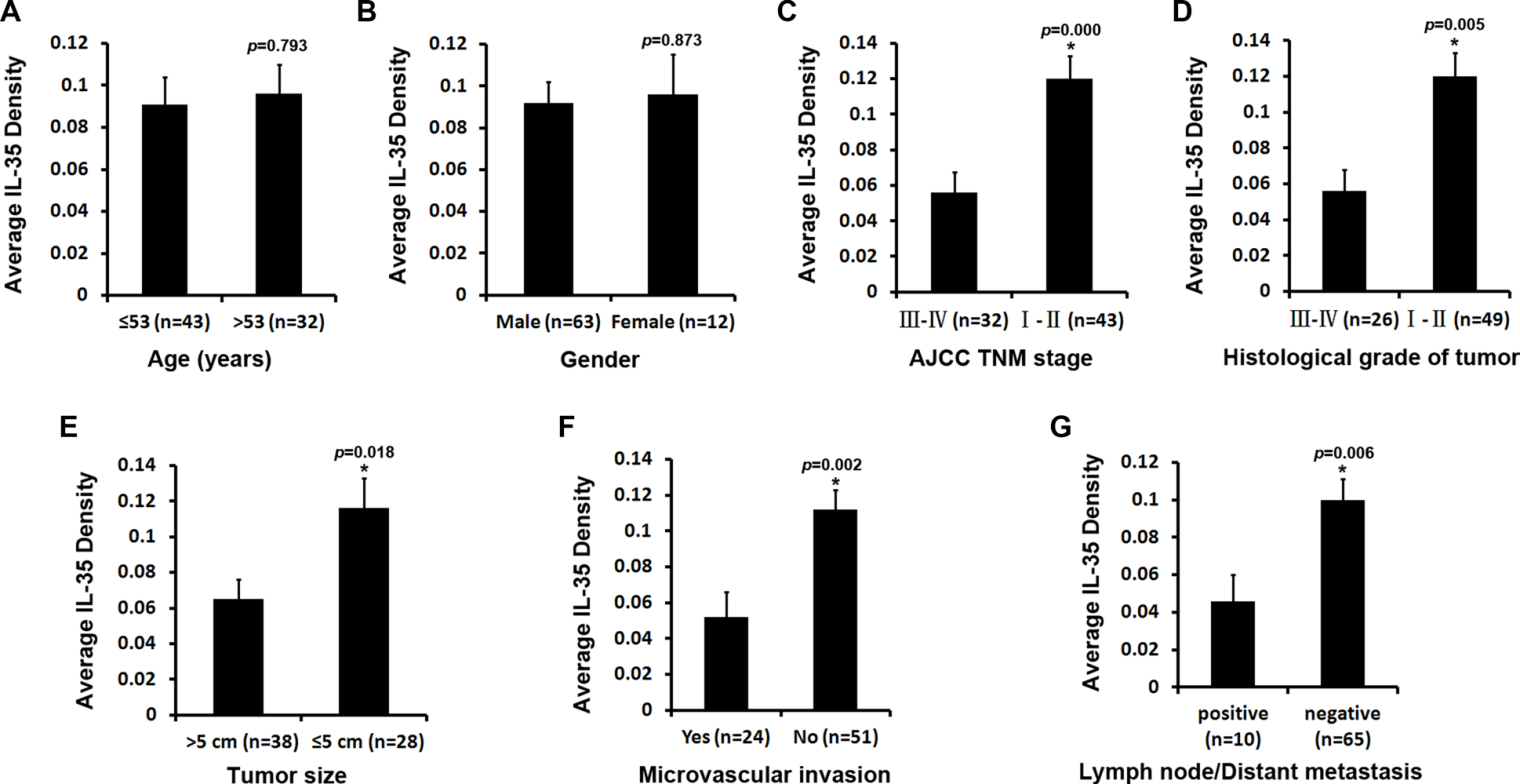
Relationship between IL-35 expression and clinicopathological features of HCC Semi-quantification of the IL-35 expression was performed by measuring the density of positive staining. Neither age (**A**) nor gender (**B**) of patients was significantly associated with IL-35 expression in tumor tissues. However, IL-35 expression was significantly lower in patients with advanced AJCC TNM stages (III-IV) compared to early stages (I–II) (**C**). Similarly, significantly poorer expression of IL-35 was observed in HCC patients with higher histological grades (**D**), larger tumor size (**E**), positive microvascular invasion (**F**) and lymph node/distant metastasis (**G**). Data are expressed as the mean ± SEM. A significant difference between the two groups is indicated by an asterisk (*, Student's *t*-test).

To further understand how IL-35 expression contributes to the progression of HCC, we investigated the relationship between IL-35 expression and the clinicopathological features of HCC patients. Neither age (*p* = 0.793, Figure 3A) nor gender (*p* = 0.873, Figure 3B) was associated with IL-35 expression in HCC patients. However, IL-35 expression was significantly lower in patients with AJCC TNM stages III-IV compared to stages I-II (0.056 ± 0.012 vs. 0.120 ± 0.013, *p* = 0.000, Figure 3C). Similarly, significantly lower expression of IL-35 was observed in HCC patients with higher histological grades (0.059 ± 0.013 vs. 0.110 ± 0.012, *p* = 0.005, Figure 3D), larger tumor size (0.065 ± 0.011 vs. 0.116 ± 0.017, *p* = 0.018, Figure 3E), positively microvascular invasion (0.052 ± 0.014 vs. 0.112 ± 0.011, *p* = 0.002, Figure 3F) and lymph node/distant metastasis (0.046 ± 0.014 vs. 0.100 ± 0.011, *p* = 0.006, Figure 3G). This result suggests that the decreased expression of IL-35 in tumor tissues might contribute to the progression of HCC.

### Over-expression of IL-35 in HepG2 cells significantly upregulated HLA-ABC and CD95, reduced the activities of MMP-2 and MMP-9, and decreased the abilities of cell migration, invasion, and colony formation

**Figure 4:**
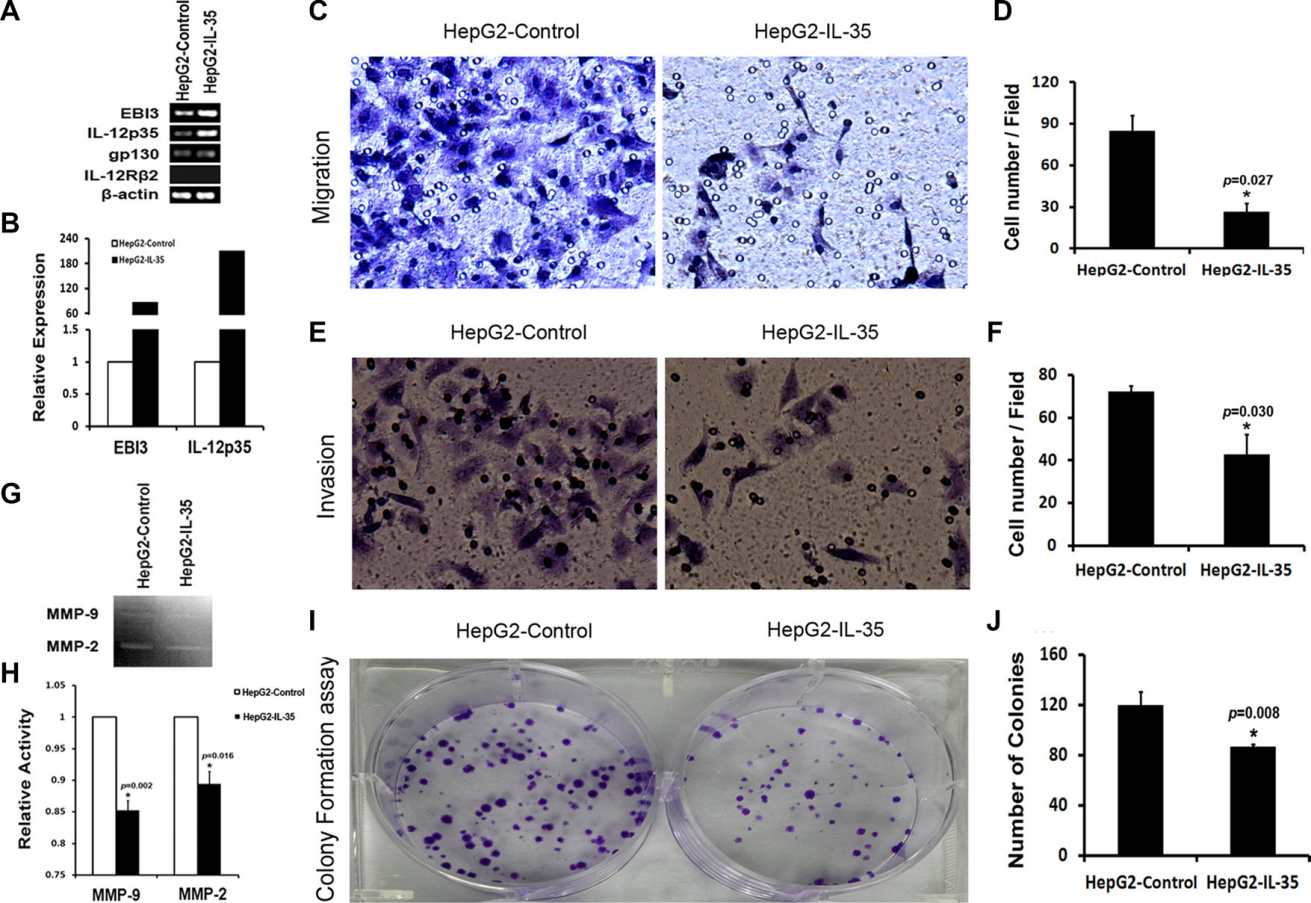
Over-expressing IL-35 in HepG2 cells reduced the activities of MMP-2 and MMP-9, inhibited cell migration, invasion and colony formation *in vitro* Detection of IL-35 or IL-35R expression was performed in established HepG2 cells stably transfected with IL-35-Fc or Fc expression vector by RT-PCR (**A**) and quantitative real-time PCR (**B**). Cell migration and invasion abilities were determined by a transwell chamber assay. Gelatin zymography assay was conducted to detect active MMP-2 and MMP-9 secretion in cells. The capacity of colony formation was determined by a colony formation assay. The cells that migrated or invaded were stained with 1% crystal violet solution and visualized by microscopy. Representative images for cell migration (× 400, C), invasion (× 400, E), gelatin (**G**) and colony formation (**I**) in the cells with or without IL-35 over-expression. Quantitative data (D, F, H, J) represent the mean ± SD of three independent experiments performed in triplicate. A significant difference between cells is indicated by an asterisk (*, Student's *t*-test). HepG2-Control: HepG2 cells stably transfected with the Fc expression vector. HepG2-IL-35: HepG2 cells stably transfected with the IL-35-Fc expression vector.

**Figure 5:**
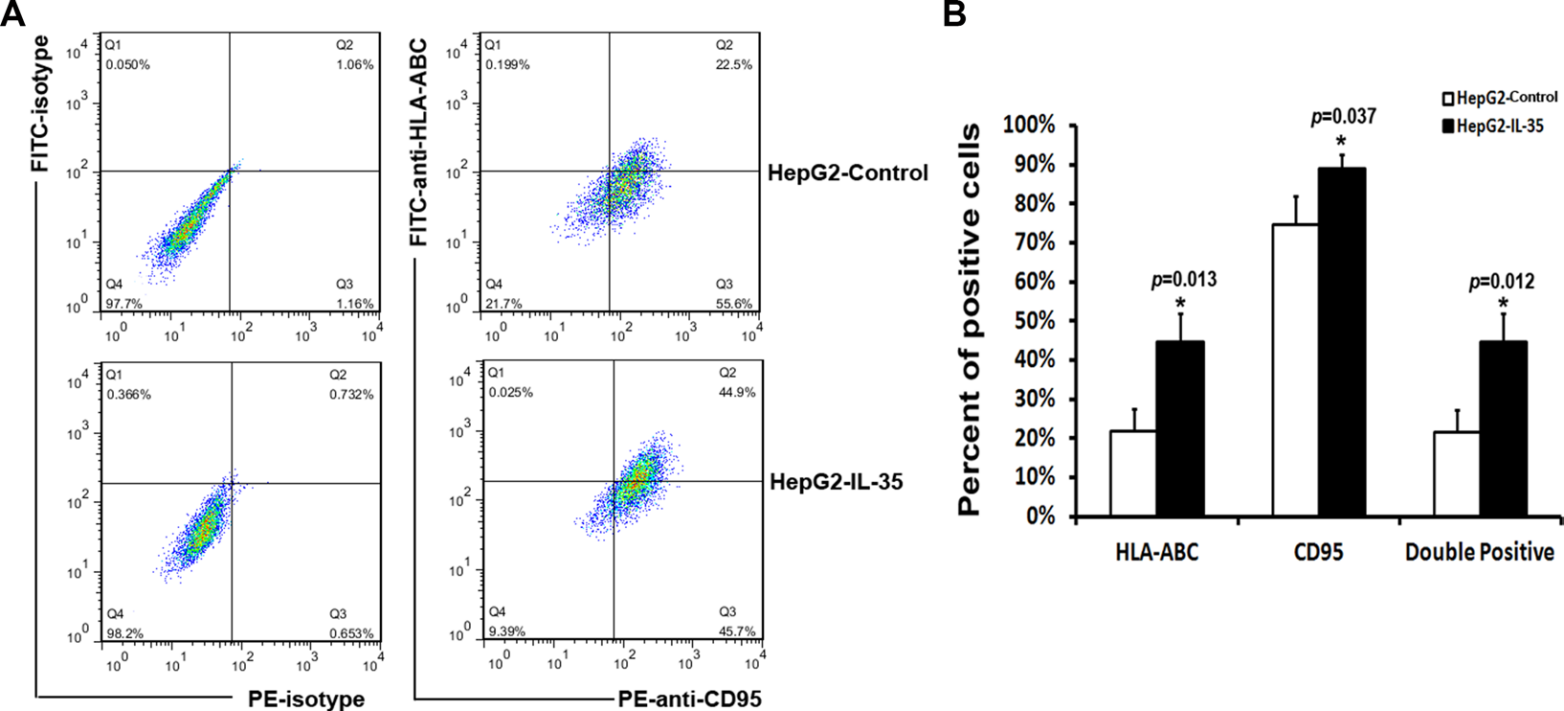
Flow cytometry analysis of the expression of HLA-ABC and CD95 in HepG2 cells (**A**) Representative dotgrams showing the expression of HLA-ABC and CD95 in HepG2 cells over-expressing IL-35 and control cells. (**B**) The percentages of HLA-ABC^+^ cells, CD95^+^ cells and HLA-ABC^+^/CD95^+^ HepG2 cells with IL-35 over-expression and control cells. Columns represent the mean ± SD of three independent experiments performed in triplicate. A significant difference between cells is indicated by an asterisk (*, Student's *t* test). HepG2-Control: HepG2 cells stably transfected with the Fc expression vector. HepG2-IL-35: HepG2 cells stably transfected with the IL-35-Fc expression vector.

Using established HepG2 cells stably transfected with IL-35-Fc or Fc expression vector [27] (Figure 4A–4B), we sought evidence that IL-35 expression in HCC cells was directly associated with the progression of HCC. We observed that the over-expression of IL-35 in HepG2 cells significantly decreased cell migration (26.73 ± 5.76 *vs.* 85.17 ± 11.17, *p* = 0.027, Figure 4C–4D) and invasion potency (42.94 ± 9.25 *vs.* 72.18 ± 2.65, *p* = 0.030, Figure 4E–4F). MMP-2 and MMP-9, two of the main proteolytic enzymes for degrading the extracellular matrix (ECM) and the basement membrane, are known to be critical for tumor metastasis. Gelatin zymography assay showed that IL-35 over-expression in HepG2 cells significantly reduced the activities of MMP-2 (*p* = 0.016) and MMP-9 (*p* = 0.002) (Figure 4G–4H). Furthermore, a colony formation assay showed that HepG2 cells with IL-35 over-expression grew significantly fewer colonies of smaller size compared to HepG2 cells without IL-35 over-expression (86.33 ± 2.52 *vs.* 119.33 ± 11.37, *p* = 0.008, Figure 4I–4J). To further elucidate the underlying mechanism, we examined whether IL-35 over-expression changed the expression levels of HLA-ABC and CD95 in HepG2 cells. We found that IL-35 over-expression also upregulated the expression of HLA-ABC and CD95 (*p* < 0.05 *vs.* controls) (Figure 5). These results supported that the decreased expression of IL-35 in tumor tissues might contribute to the progression of HCC, likely through anti-tumor immune mechanisms.

## DISCUSSION

In this study, we tried to understand the role of IL-35 on the progression of HCC, one of the most frequent cancers with high lethality worldwide. We found that the expression levels of IL-35 were significantly higher in the peri-tumoral liver tissues than in the tumor tissues. Furthermore, lower IL-35 expression in tumor tissue was observed in HCC patients with AJCC TNM stages, worse histological grades, larger tumor sizes, and histological identification with microvascular invasion and lymph node/distant metastasis. These data indicated that decreased IL-35 might be negatively associated with the prognosis of HCC. In contrast, previous studies showed that the expression of IL-35 increased with advancing tumor stages in colorectal cancer [21] and nasopharyngeal carcinoma [22].

Tumor metastasis and growth is a complicated process involving multiple steps [28], and cell migration, invasion and resistance to cell apoptosis are essential features of the metastatic process [29–30]. Our previous study [27] has revealed that the over-expression of IL-35 to HepG2 cells enhances apoptotic sensitivity, but little is known about the role of IL-35 expression in the metastasis of HCC. In the present study, we confirmed that the over-expression of IL-35 in HepG2 cells reduced the activities of MMP-2 and MMP-9, significantly decreased cell migration, invasion and single cell colony formation abilities, suggesting a poor potency for metastasis.

The molecular mechanism on IL-35 regulating the progression of HCC remains unclear. IL-35 is well defined as an anti-inflammatory and immune inhibitory cytokine. The tumor can produce a series of mechanisms to evade attack from the immune system, such as the down-regulation of MHC class I and CD95 molecules on the surface of tumor cells. The over-expression of IL-35 in HepG2 cells significantly upregulated HLA-ABC and CD95, suggesting an anti-tumor activity of IL-35 expression in HCC. Recently, Wang et al. [23] has reported that IL-35 over-expression in plasmacytoma and melanoma cells does not alter the expression of MHC class I and shows very limited effects on cell growth *in vitro*, but stimulates tumorigenesis in both immunocompetent and Rag1/2-deficient mice through the suppression of tumor immunity. Vignali and colleagues show that Treg cell-derived IL-35 promotes tumorigenesis by contributing to T cell exhaustion and thereby limiting anti-tumor immunity [31].

The reason for the divergent results between our study and previous reports remains unclear. IL-35 signals through IL-12Rβ2: gp130 heterodimer or homodimers of each chain, activating transcription factors Stat1 and Stat4 [32]. HepG2 cells express one of IL-35R subunit gp130, but not another subunit IL-12Rβ2 [27]. Therefore, the effects of IL-35 may different greatly depending on the expression, distribution and utilization of receptor complex in different cell types.

In summary, our data provided evidence that IL-35 was involved in the progression of HCC, probably by directly inhibiting cell activities and indirectly modulating the immune system. IL-35 may serve as a novel prognostic biomarker and therapeutic targets for HCC.

## MATERIALS AND METHODS

### Patients and HCC tissue samples

This study was performed on surgical samples from 75 Chinese patients who underwent precise hepatectomy with pathological confirmation of primary HCC. All samples were obtained following informed consent according to an established protocol approved by the Ethic Committee of Capital Medical University. Clinical information, such as age, gender, presentation and pathological findings including tumor size, microvascular invasion, lymph node status, distant metastasis, histological grade of tumor, and tumor stage, were obtained from the original pathology reports. The data do not contain any information that might lead to the identification of the patients.

### Tissue microarray (TMA) and immunohistological staining

A pair of samples (1.5 mm in diameter) taken from the primary tumor tissue and peri-tumoral liver tissue (approximately 15 mm distance from tumor) of each HCC patient was used for the construction of a TMA (in collaboration with Shanghai Xinchao Biotechnology Company, Shanghai, China) as previously described [33]. The constructed TMA was cut into 4 μm thick sections for immunohistological staining. Briefly, TMA slides were deparaffinized, rehydrated through graded alcohol, and washed with PBS. Antigen retrieval was performed by microwave-heating sections in 10 mM sodium citrate buffer (pH 6) for 10 minutes. After quenching of endogenous peroxidase activity and blocking of nonspecific binding, mouse anti-human IL-35 monoclonal antibodies (Imgenex, San Diego, CA) were added at a specified dilution (10 μg/ml), after which slides were incubated at 4°C overnight. HRP conjugated anti-mouse IgG was used as a secondary antibody to detect anti-human IL-35 antibody binding. Chromogenic immunolocalization was performed by exposure to 3, 3′-diaminobenzidine (DAB) substrate. Slides were counterstained with hematoxylin before dehydration and mounting.

The density of positive staining was measured using a computerized image system composed of a Leica CCD camera DFC420 connected to a Leica DM 6000B microscope (Leica Microsystems Imaging Solutions Ltd, Cambridge, United Kingdom). Under high-power magnification (× 400), photographs of four representative fields were captured by the Leica QWin Plus v3 software. Identical settings were used for each photograph. The IL-35 density was measured by the Image-J software (Media Cybernetics Inc, Bethesda, MD, USA). The integrated optical density of the positive staining of IL-35 in each photograph was measured, and its ratio to total area of each photograph was calculated as the IL-35 staining density.

### Cell culture

HepG2 cells with established stable IL-35 over-expression and control cells were obtained as previously described [27]. These cells were cultured in high glucose DMEM medium (Hyclone, USA) supplemented with 10% fetal bovine serum (FBS, Hyclone, USA), 100 units of penicillin/ml (Hyclone, USA), and 100 ng of streptomycin/ml (Hyclone, USA). The cells were incubated at 37°C in an atmosphere of 5% CO2.

### RT-PCR and quantitative real-time PCR

Total RNA was isolated using TRIzol Reagent (Invitrogen, USA), then reverse transcribed into cDNA using the RNA-to-cDNA Kit (Genestar, China). The primer sequences used in this study are shown in Table 2. The PCR products were amplified using the following thermal cycles parameters: 95°C for 30 s, 60°C for 30 s and 72°C for 30 s. The PCR amplicons were visualized using a 2% agarose gel containing ethidium bromide. The relative gene expression levels were determined using quantitative real-time PCR and the SYBR Green labeling method in a Rotor-Gene Q continuous fluorescence detector (Qiagen, Germany), and the relative gene expression levels were calculated using the ΔCt method, which was normalized to the endogenous *β*-actin as a control. The data are expressed as n-fold relative to the control.

**Table 2:** The Primer sequences of the target genes

Gene	Forward sequence (5′–3′)	Reverse sequence (5′–3′)	Product size (bp)
*EBI3*	GCTTCGTGCCTTTCATAACAG	GCTCCCACTGCACCTGTA	102
*IL-12p35*	ACATGCTGGCAGTTATTGATGA	TGAAGAAGTATGCAGAGCTTGAT	127
*gp130*	GCAGTTTGTGTGCTAAAG	AATGTTGCAAGTGAGCTG	180
*IL-12Rb2*	CAAGAGAGGCGATGTGAC	TGAGAATTGAGGGAGTGG	180
β-*actin*	GCATCCTCACCCTGAAGTAC	TGATCTGGGTCATCTTCTCG	180

### Transwell chamber assay

A transwell chamber assay was used to evaluate the capacity of cell migration and invasion using two-chamber plates with a pore size of 8 μm according to the manufacturer's instructions (Corning, USA). DMEM with 10% fetal bovine serum was added to the lower chamber as a chemoattractant. For the invasion assay, 3 × 10^4^ cells were seeded in serum-free medium in the upper chamber coated with extracellular matrigel (ECM) on the transwell filter inserts, while the cell migration assay performed using a coat without ECM (Corning, USA). After incubation at 37°C for 48 h, the cells in the upper chamber and on the matrigel were mechanically removed with a cotton swab, and the cells on the outer surface of the membrane were stained with 1% crystal violet (Sigma, USA). The migrating and invading cells were examined, counted, and photographed by digital microscopy (Leica Microsystems Imaging Solutions Ltd., Cambridge, United Kingdom). Five fields of view per filter were counted; the fields were randomly chosen from the top, bottom, left, right, and center positions of each filter.

### Gelatin zymography assay

A gelatin zymography assay was conducted to evaluate the influence of IL-35 on the activities of matrix metalloproteinase (MMP-2 and MMP-9). 8 × 10^5^ cells were seeded into six-well plates, cultured with serum free medium for 24 h. At the end of incubation, 30 μL of culture supernatant was mixed with sample buffer and resolved on a 10% SDS-PAGE under non-reducing conditions. The gel was co-polymerized containing 1 mg/mL of gelatin (Sigma, St. Louis, USA). Gel was washed twice for 30 min with renaturation buffer (2.5% Triton X-100) at room temperature before incubation in the incubation buffer (50 mM Tris-HCl pH7.5, 200 mM NaCl, 10 mM CaCl2, 1 μM ZnCl2) at 37°C for 24 h. Thereafter, gel was stained for 2 h in 0.5% coomassie brilliant blue R-250 and then de-stained. White bands were observed against a blue background after de-staining, indicating gelatinolytic activity of MMP-2 and MMP-9. The activities of the MMP-2 and MMP-9 bands were quantified using densitometry by the Image-J software (Media Cybernetics Inc, Bethesda, MD, USA).

### Colony formation assay

HepG2 cells over-expressing IL-35 and control cells were collected, and 300 cells were seeded into six-well plates for 2 weeks. Colonies were fixed with 4% (v / v) paraformaldehyde for 20 min and stained with 1% crystal violet (Sigma, USA) for 30 min. The number of colonies was measured.

### Flow cytometry

Cells were stained with FITC-labeled antibody against human HLA-ABC and PE-labeled antibody against human CD95(Miltenly). The appropriate isotype controls were used as negative controls. Analytical flowcytometry was performed with a BD FACSAria flow cytometer (BD Biosciences, USA) and the data were analyzed using the FlowJo software (TreeStar, Ashland, OR).

### Statistical analysis

Data are expressed as mean ± SEM or mean ± SD. Quantitative variables were analyzed by Student's *t*-test. All statistics were two-tailed, with a *p* value < 0.05 considered statistically significant. Analyses were performed using the SPSS software package (version 11.5) (SPSS Inc, Chicago, IL).
